# Case Report: Reintroduction of winged infusion sets to needle syringe programs - advancing equity in harm reduction

**DOI:** 10.3389/fpsyt.2026.1741947

**Published:** 2026-06-10

**Authors:** Caroline James, Vendula Belackova, Wendy Machin, Olivia Thackeray, Louisa Jansen, Danielle Resiak, Nina Hutchinson, Ian Anderson, Nicholas Rich, Phillip Read

**Affiliations:** 1 Kirketon Road Centre, South Eastern Sydney Local Health District, Sydney, NSW, Australia; 2 Social Policy Research Centre, Faculty of Arts and Architecture, University of New South Wales (UNSW), Sydney NSW, Australia; 3 The Kirby Institute, University of New South Wales (UNSW) Australia, Sydney, NSW, Australia

**Keywords:** drug policy, harm reduction, methadone injection, needle and syringe program (NSP), people who inject drugs (PWID), health service equity, winged infusion sets

## Abstract

**Background:**

In January 2023, following a 20-year ban, the New South Wales (NSW) Needle and Syringe Program (NSP) Guidelines permitted NSPs to provide winged infusion sets (‘butterflies’) and larger-volume syringes (‘barrels’) commonly used for injecting methadone liquid intravenously.

**Approach:**

The Kirketon Road Centre (KRC) integrated the distribution of ‘butterflies and barrels’ (referred to as ‘butterfly packs’) into its service delivery in April 2023 and developed resources in collaboration with the Uniting Medically Supervised Injecting Centre (MSIC) and the NSW Users and AIDS Association (NUAA). This case report presents an audit of service delivery and self-reported client outcomes following the introduction of ‘butterfly packs’.

**Outcomes:**

Since April 2023, KRC has been consistently supplying ‘barrels’ and ‘butterflies’ to clients who inject methadone. The proportion of NSP visits by clients who reported methadone as the last drug injected increased significantly after the program’s introduction (from 2.2 to 4.0 methadone-related attendances per month), and so did the amount of corresponding injecting equipment and verbal education sessions. Among 18 survey respondents, the majority reported improved health or safety since accessing butterfly packs (89%).

**Conclusion:**

Equipment for intravenous methadone administration can be safely and efficiently distributed by NSP programs. Access to harm reduction services should be based on vulnerability and need, with services tailored accordingly. The policy reversal allowing ‘butterflies and barrels’ and service innovations such as KRC’s program have addressed a longstanding gap, advancing equity in harm reduction for people who inject methadone in NSW.

## Introduction

Methadone is a long-acting pharmaceutical opioid used in the treatment of opioid use disorder (OUD). It is typically formulated as a syrup or liquid intended for oral consumption (1). People may seek to inject methadone for various reasons, such as faster relief of opioid withdrawal (2), perception of more pleasurable effects from injecting as opposed to ingestion, due to a preference of injecting as a route of opioid administration, or because of any adverse effects of methadone while ingested (3, 4).

Injecting methadone is discouraged by opioid treatment programs due to concerns about patients’ stability (5) as well as because of the harms from methadone injecting (heightened risk of overdose or the risks of intravenous administration in itself) (1). Methadone injecting increases the risk of vein or tissue swelling and necrosis due to the viscosity of the solution, pulmonary embolism when foreign particles enter the lungs, and of cardiac infections (6). Biodone formulation, compared to more diluted methadone syrup, may have a lower risk profile when injected (5). Clinical studies have explored the use of injectable methadone under clinical supervision for people who have not stabilized on oral methadone (7).

People who inject methadone may use the drug prescribed to them for oral use, or use methadone obtained via friends and the illicit drug market (8). In New South Wales (NSW), Australia, between 2023 and 2024, 12% of people who inject drugs reported recently using non-prescribed methadone — the lowest recorded proportion since monitoring began in 2003. The highest rate was recorded in 2009, when approximately one-third of people who inject drugs reported using non-prescribed methadone (9). During the same period, the number of methadone-related deaths per 100,000 in NSW increased from 0.4 per 100,000 population in 2003 to 0.7 in 2023, with a peak in 2018 of 1.4 respective deaths (10). At the same time, research from Australia has confirmed the risk of opioid overdose to decrease by more than half among people in opioid agonist treatment with methadone or buprenorphine (11, 12).

Methadone (and particularly syrup’s) viscous preparation makes it unsuitable to inject and diluting with water is seen as a better way of administration. To inject diluted methadone, large ‘barrel’ syringes (10 mL, 20 mL or 25 mL) may be required, potentially in combination with winged infusion sets, also known as ‘butterflies’ that allow syringes to be changed without needing to remove the needle from the injection site (13).

In 1999, in response to nearly one third of people who used heroin reporting recent injection of methadone (14), the government prohibited public NSPs from distributing equipment for methadone injection, although the equipment remained available for purchase via private pharmacies (13). Under this policy, people who inject methadone faced greater barriers to receiving injecting equipment than people who inject other drugs. This has created an issue under the lens of health equity, as all people should be valued equally when it comes to health (15) and harm reduction programs (16).

Opinion on the ban at the time was divided, with some experts suggesting that an effective policy to reduce methadone injecting in New South Wales may have been to, instead, reduce the number of take-away doses of methadone (1). A study has shown that following the ban of ‘butterflies and barrels’ from NSPs, people who inject methadone increased reuse of their injecting equipment (13).

## Approach

### Re-introducing ‘butterflies and barrels’ into NSPs: service delivery model

In January 2023, the NSW Needle and Syringe Program (NSP) Guidelines were updated allowing NSW NSPs to once again provide winged infusion sets (‘butterflies’) and larger volume syringes (‘barrels’) for distribution across NSW. Kirketon Road Centre (KRC), in collaboration with other providers, innovated services to re-introduce the equipment.

KRC is located in inner-city Sydney and provides comprehensive health and support services, ranging from Needle Syringe Program (NSP), nurses, doctors (at Kings Cross), counselling, and other supports. Within the NSP and the associated community room, clients can access injecting equipment, harm reduction education, take home naloxone training, nicotine replacement therapy, and testing for hepatitis C and HIV (dried blood and point of care testing). Referrals may be made to the adjunct health and counselling services on a per needs basis to tackle various health issues, testing for sexually transmissible infections, hepatitis C or HIV treatment, vaccinations, opioid treatment program, psychiatric registrar, or psycho-social support including housing. KRC implemented the distribution of ‘butterfly packs’ into its service delivery in April 2023; each include 10 butterflies, twenty 10 ml barrels, and a peer-to-peer resource (flier) developed by the NSW Users and AIDS Association (NUAA). The flier is also available online at https://nuaa.org.au/safer-using among other harm reduction resources (17).

Together with the Uniting Medically Supervised Injecting Centre (MSIC), KRC co-produced a comprehensive medical training video and collaborated to deliver an Online Masterclass for NSP workers about methadone injecting.

The packs containing equipment and fliers have been distributed via KRC’s primary NSPs, outreach services, and some Opiate Agonist Treatment (OAT) programs across the South Eastern Sydney Local Health district (SESLHD). Informing clients about the availability of ‘butterflies and barrels’ has been done on a one-on-one basis when trained staff approached clients who have previously reported methadone as the last drug they injected. This approach was chosen over open advertising. The winged infusion sets are relatively costly compared to other NSP equipment and KRC aimed to target people who currently inject methadone. An additional piece of equipment that is used for filtering methadone prior to injecting (“*wheel filter*”) has been available via parter community organizations, NUAA and MSIC, as it has been cost-prohibitive to provide at KRC.

For disposal, all NSP clients are being asked to use puncture-resistant containers for used injecting equipment and return them to the NSP or to any other “community sharps bin” available in the area (placed nearby dispensing machines with NSP equipment and at community health centers). Clients accessing ‘butterfly packs’ are given larger containers that are best suited for bulkier items like winged infusion sets. Smaller containers or some public disposal bins (e.g. in public toilets or hospitals) may have smaller chute openings, making them more suitable for compact injecting equipment than for ‘butterflies’.

This case study aimed to describe the outcomes of KRC’s ‘butterfly program’ in terms of needle-syringe service delivery and impact on clients.

### Data audit

We conducted an audit of ‘butterflies’ and ‘barrels’ equipment at KRC by reviewing all respective orders of since February 2022 (to commence dispensing in April 2023). We also checked KRC’s records of unsafely discarded injecting equipment retrieved from public spaces to determine consequent unsafe disposal of ‘butterflies’ and ‘barrels’. Subsequently, we reviewed KRC’s records of NSP distribution during a three-year period 1 April 2022–30 April 2025 (this period covered 13 months prior to the program implementation in April 2023 and 23 months after). The timeframe was selected to capture a period before and after the ‘butterfly’ program was introduced and was determined by availability of data from KRC’s NSP database.

From the NSP database, we reviewed characteristics of the people who indicated that the last drug they used was methadone, and the number of dispensing occasions, equipment dispensed, and services/referrals provided to them. No identifying information has been collected by the NSP program (being an anonymous, drop-in service); as such, even though the clients may have been linked to additional health care at KRC or elsewhere, the number of services/referrals was the only objective information available regarding client outcomes.

Units of injecting equipment that were ordered (and dispensed) were organized into quarterly sums, to avoid any bias potentially introduced by looking at uneven length of time before and after; records of NSP dispensing were organized as monthly totals and averages (per occasions of service) before and after the beginning of the ‘butterfly program’. We conducted a descriptive analysis of orders of ‘butterfly equipment’ and reviewed records of discarded equipment.

### Client survey

Finally, in October 2024, NSP staff designed a brief, fit-for-purpose survey, encompassing for informal feedback from the clients of the ‘butterfly’ program during their interactions with staff since program inception. The survey was designed as a tool used to provide timely evaluation of the new service. No socio-demographics were included in the survey.

Seven structured questions were asked about how clients learnt about the program (“NSP site”, “outreach”, “word of mouth”, “flyer”), frequency of using ‘butterflies and barrels’ (ranging from “every time they inject” to “often”, “occasionally”, “rarely”, and “never”), subjectively if they noticed an improvement in their safety and health following the program introduction (“yes”, “no”, “not sure”), whether and how they received harm reduction information (“yes, face to face”, “yes, through fliers”, “no”, or “other”), their satisfaction with the availability of butterflies (“very satisfied”, “satisfied”, “neither satisfied nor dissatisfied”, “dissatisfied”, or “very dissatisfied”), whether the availability of ‘butterflies’ affected their decision to visit NSPs (“yes, increased visits”, “no, hasn’t affected visits”, “not sure”), and whether they had any concerns about the safety or disposal of the equipment (“yes”, “no”, “not sure”). Two of the structured questions offered a space for open-ended answers (in relation to improvements in health or safety, and in relation to receiving harm-reduction information), and one open-ended question asked respondents to suggest any improvements to the ‘butterfly’ program the clients would like to see.

SESLHD Research and Governance Office assessed this project on 19 May 2023 as not raising any ethical concerns. The survey was distributed by NSP workers and peers at KRC’s fixed locations and outreach. Everyone who was accessing the ‘butterfly’ equipment from KRC at the time of the survey was asked to complete it on paper when they were receiving the NSP service. All clients who were approached agreed to participate.

We conducted descriptive analyses of the survey data to summarize patterns of program use and perceived impacts. Given the small sample size and binary outcomes, we used Fisher’s exact tests to explore whether increased NSP attendance following the introduction of the butterfly program was associated with key client-reported factors, including frequency of butterfly equipment use, perceived improvements in health or safety, and satisfaction with equipment availability. These comparisons aimed to assess whether perceived benefits were more commonly reported among participants with higher levels of program use, while avoiding over-interpretation of inferential findings in this exploratory, service-based evaluation. Subsequently, we summarized open-ended client’s comments into main themes.

## Outcomes

**Figure 1:**
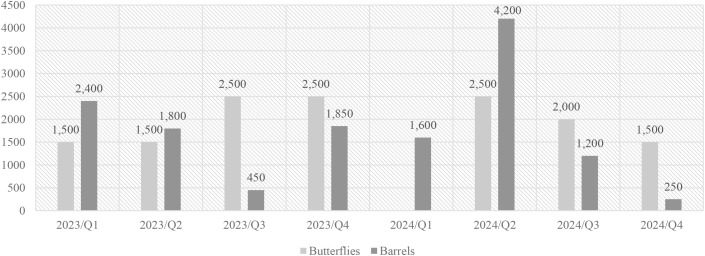
Audit of butterflies and barrels – orders by quarter between 1 April 2023 and 30 December 2024.

Over the audit period, KRC supplied 13,750 ‘barrels’ and 15,500 ‘butterflies’ to their clients, see **Figure 1** for details. This has mirrored a steady demand from clients who inject methadone (see below). Since the re-introduction of the ‘butterfly program’, KRC has collected 63,593 units of discarded injecting equipment in public spaces; ‘butterflies’ were not identified on the street as individual unsafely discarded items.

Between 1 April 2023–30 April 2025, there were 125 dispensing occasions of NSP equipment at KRC to people who indicated the last drug they injected was methadone (of a total 18,383 visits made to the program). Among methadone-related visits, two (1.6%) were to people under the age of 25 years, 22 were to people aged 25–35 years (17.6%), and majority were >35 years old (81%); on 77 occasions, these individuals were male (62%).

The number and proportion of NSP clients who reported that the last drug they injected was methadone were low overall (<1%) but showed a statistically significant increase after April 2023 when the butterfly program was introduced. The mean monthly number of visits by people whose last used drug was methadone rose from 2.2 to 4.0 (*p* < 0.001). This corresponded to an increase in the mean proportion of NSP visits related to methadone from 0.6% to 0.8% (*p* < 0.001). The mean number of verbal education sessions provided to these clients also increased substantially, from 1.5 to 3.9 (*p* < 0.001).

NSP dispensing patterns also changed significantly. The mean monthly amount of combined needle–syringe equipment distributed to people reporting last drug used methadone increased from 150.3 to 387.8 (*p* < 0.001), while needles-only and syringes-only distribution decreased markedly (from 22.4 to 1.3, and 22.5 to 2.4, respectively; *p* < 0.001 for both). On a per-visit basis, the mean amount of combined equipment (including ‘butterfly packs’) rose from 67.6 to 87.0 (*p* < 0.001), whereas needles-only and syringes-only averages declined (from 7.5 to 0.3 and 9.4 to 0.6, respectively; *p* < 0.001 for both). See **Table 1** for details.

**Table 1:** Records of dispensed NSP equipment.

	Before 30 April 2023 (n=13months, encompassing for 4,984 NSP visits) Mean (SD)	After 30 April 2023 (n=24 months, encompassing for 13,399 NSP visits) Mean (SD)	Mean difference	t-value	p-value
Mean monthly number of visits to NSP when last drug used was methadone	2.2 (1.1)	4.0 (2.4)	+1.8	51.14	<0.001
Mean proportion of all visits to NSP when last drug used was methadone (%)	0.6 (0.3)	0.8 (0.6)	+0.3	31.16	<0.001
Mean monthly no of verbal education to NSP attendees who last used methadone	1.5 (1.1)	3.9 (2.4)	+2.4	67.97	<0.001
Per month: mean amount of dispensed equipment to people who last used methadone					
Needles & syringes combined	150.3 (100.3)	387.8 (322.1)	+237.6	51.03	<0.001
Needles only	22.4 (56.8)	1.3 (4.1)	−21.0	−39.84	<0.001
Syringes only	22.5 (57.0)	2.4 (7.5)	−20.1	−37.33	<0.001
Per service occasion: mean amount of dispensed equipment to people who last used methadone					
Needles & syringes combined	67.6 (29.7)	87.0 (40.1)	+19.3	30.61	<0.001
Needles only	7.5 (17.8)	0.3 (0.8)	−7.2	−43.13	<0.001
Syringes only	9.4 (26.7)	0.6 (2.2)	−8.8	−34.98	<0.001

Finally, eighteen clients completed the survey. Ten (56%) participants said they used the equipment from KRC’s program every time they injected. The majority of participants heard about the availability of this equipment from NSP site or outreach program (78%); the remainder (22%) heard about it via “word of mouth”.

All clients were either satisfied or very satisfied with the availability of the equipment. Fourteen said that the re-introduction of ‘butterfly packs’ has increased their visits to the NSP (78%). Sixteen (89%) indicated that they noticed improvements to their safety or health since they started accessing the ‘butterfly’ program, and two said they were not sure about the impact (see **Table 2**).

**Table 2:** Survey results.

	No.	Proportion	Has the availability of butterfly needles and larger barrels impacted your decision to visit our NSP sites/services?				
			Yes, increased attendance", No. (%)			No increase in attendance", No. (%)	
All participants	18	100%	14 (78%)			4 (22%)	
How frequently do you use the butterfly needles and larger barrels provided by our program?							
				No.	No. (%)	No. (%)	Fisher's exact test
Every time I inject	10	56%	Every time/often	12	11 (92%)	1 (8%)	0.083
Often	2	11%					
Occasionally/Rarely	6	33%	Occasionally/Rarely	6	3 (50%)	3 (50%)	
How satisfied are you with the availability of butterfly needles and larger barrels at our distribution sites?							
Very satisfied	16	89%	Satisfied/very satisfied	16	13 (81%)	3 (19%)	0.405
Satisfied	2	11%					
Neutral/Dissatisfied/Very Dissatisfied	0	0%	Neutral/dissatisfied	2	1 (50%)	1 (50%)	
Since the distribution of butterfly needles and larger barrels began, have you noticed an improvement in your safety and health?							
Yes,	16	89%	Yes	16	13 (81%)	3 (19%)	0.405
No/not sure	2	11%	No/not sure	2	1 (50%)	1 (50%)	

Increased attendance was most pronounced among participants who used butterfly equipment frequently (every time or often), where 92% reported increased attendance compared with 50% among those using the equipment occasionally or rarely (Fisher’s exact p = 0.083). In contrast, although most participants reported improvements in health and safety (81%) and high satisfaction with equipment availability (81%), these outcomes were similarly distributed among participants with and without increased NSP attendance, with no evidence of association (both p = 0.405).

Clients who participated in the survey explained how accessing the program may have improved their health. Firstly, they noted availability of ‘butterflies’ at NSPs has increased their use of clean equipment: “*I don’t have to reuse butterflies because I can afford not to*”, “*it has stopped me to reuse old ones until they are blunt and do more damage than good*”. They also provided other examples of improving their injecting practice (“*Good to be able to dilute and infuse more appropriately*”). One client reported they did not know about availability of hepatitis C testing at NSPs until they started coming to the NSPs to get ‘butterfly packs’.

There were multiple suggestions for improvement from the clients, many pertaining to further increasing availability (“*open on weekends”, “add to vending machines”, “larger quantities*”) and types of equipment provided (suggestions were to include “*wheel filters”, “blue filters”, “provide steri-cups and filters rather than spoons*”, or “*change to non-screw in barrels*”). Two participants had concerns about the safety of disposal (“*there needs to be more disposal points*”).

Overall, clients were grateful for the service and complimented the staff providing the equipment. A few participants appreciated the program was a shift towards harm reduction policy and practice: “*Another step forward in uniform inclusion and assistance to more people who inject drugs*”. One client stated that “*NSPs have finally included people who use butterflies in the community*”.

## Discussion

Our study suggests that removing the ban on methadone-injecting ban from public NSPs may have facilitated positive behavior changes – ranging from a (self-assessed) decrease in re-using NSP equipment, to an increase in NSP attendance (potentially among people who used ‘butterfly’ equipment frequently), or perceived improvements in safety and health. This complements the findings of previous research which documented an immediate increase in re-using equipment from methadone injection following the ban (13). Distribution of ‘butterflies and barrels’ has provided an opportunity to include people who inject methadone in the NSP community and as such, link them with a broad range of services that are available via NSPs.

### Innovation in service delivery and access to safer equipment

Demand for this equipment has been managed without significant resource pressure at KRC, despite the equipment being relatively costly. Since the re-introduction of the ‘butterfly program’, there has been no evidence of this equipment being unsafely discarded in public spaces. To expand the ‘butterfly’ program, KRC would like to provide additional bacterial filtering equipment with the butterfly packs (as requested by its clients in the survey) but we noted these are a rather expensive addition to standard equipment provision, and we refer clients to partner community organizations where these can be obtained. Increasing ‘butterfly’ distribution through secondary NSP networks, and via automatic dispensing machines is under consideration.

The observed increases in methadone-related NSP visits and verbal education delivery suggest that the Butterfly Program effectively enhanced engagement with clients whose last drug used was methadone. The shift from single-item distributions (needles or syringes only) to combined equipment (butterfly packs) has aligned with the program’s harm reduction goals to facilitate safer administration of drugs. The significant increase in education sessions indicates that beyond distributing equipment, the ‘butterfly’ program has managed to promote safer practices and support informed decision-making among clients, including better access to testing and care for blood-borne viruses or to free take home naloxone.

### Trends in methadone misuse in NSW

Despite the rise in engagement and NSP distribution among people who inject methadone, this client group still represents a relatively small proportion of visits to KRC (<1%). In surveys among people who inject drugs, 1-2% indicate methadone as the drug injected most often (18) and 4-5% as last drug injected (19). The proportion at KRC may increase further when availability of ‘butterflies’ at NSPs becomes common knowledge in the community. Also, compared to surveys, there are limitations to KRC’s NSP attendance data, such as lack of information on the number of unique individuals, only one drug type being queried at each visit, or type of equipment (‘butterfly packs’) not directly recorded in KRC’s NSP database. The NSP data may also reflect on inherent differences in NSP attendance patterns between those injecting methadone vs other drugs (e.g. people who inject methadone doing so less frequently than those who inject heroin (2)). Also, at KRC’s NSP, a substantial proportion of the denominator (no. of all visits) is made up of people who inject steroids or other performance enhancing substances; this was approximately 15% over the audit period compared to <1% in the national surveys, potentially shifting the proportion attributed to any illicit drug in the NSP data downwards relative to the surveys.

Concerns may remain about what the impact of removing the ban on butterfly equipment will have on rates of methadone injecting, considering there has been a steady decrease in methadone injecting across available indicators of methadone misuse over the past 20 years. Our study, despite being indicative of greater engagement among this client group, does not provide insight into the trends in the broader community. Nationally, among people who inject drugs, the proportion of people who reported methadone as the drug they injected most often has decreased from 8% in 2008 to 1-2% in 2022 – 2024 (18). Similarly in NSW, according to the Australian National NSP survey (19), methadone as the drug last injected decreased from 10% of NSP attendants reporting this in 2007 to 4-5% in 2020 – 2022. When it comes to any non-prescribed use of methadone (irrespective of the route of administration), there has also been a gradual decrease in Australia since 2008, from about 30% of people who inject drugs reporting its misuse within the past 6 months to less than 10% in 2022 - 2024 (20). NSW-specific data from the same study have mirrored this trend, although the levels of non-prescribed methadone use have been slightly higher (12%) than the national average across 2003 - 2023 (21). Notably, a similar decreasing trend has been recorded for non-prescribed use of buprenorphine (reported on national level only), from 23% in 2005 to about 5 – 6% in 2022 – 2024 (18). Given the consistent national and NSW-specific declines in both methadone (and buprenorphine) misuse, there is little evidence to suggest that NSW Health’s ban on ‘butterflies and barrels” was a key factor in reducing methadone injecting—nor is there evidence to assume that lifting the ban will lead to a resurgence in the trend. It should be noted that a large proportion of people reporting on their injecting drug use for the trends referenced above were recruited via needle-syringe programs, potentially introducing a downward bias if the relevance of these programs had been limited by not providing equipment for methadone use.

In contrast to the declining rates of methadone injecting and misuse presented above, methadone-related deaths have increased in NSW over the past twenty years, peaking in 2018 (22). The number of methadone-related deaths in NSW had been exceeding those related to heroin until 2016 when the ratio reversed (10). The mean number of methadone-related deaths per 100,000 population in NSW between 2003 and 2023 was 0.8, the highest from all Australian states (0.7 in Queensland, Tasmania and ACT, 0.6 in Western Australia and South Australia, and 0.4 in Queensland) (10). There were different trends across Australian states, with Tasmania/ACT and South Australia experiencing a peak in methadone-related deaths in 2005–2010 and Western Australia in 2012 – 2015. For New South Wales, Victoria, and Queensland, the peak was in 2016 – 2018. These deaths occurred irrespective of the NSW ban on methadone injecting equipment at NSPs, and despite continuous decrease in reported methadone misuse. The continued methadone-related mortality indicates there is a need to better engage people who misuse or inject methadone in harm reduction. KRC’s butterfly program, as described in this case study, has been an example of such engagement.

### Increasing equity of harm reduction services

Our study, despite small numbers, re-iterates that harm reduction programs must ensure equitable access to care (2). Exclusions may occur among groups experiencing structural inequalities (16, 23), and may be exacerbated by bans of particular injecting practices. For decades, people who inject methadone were required to purchase equipment from pharmacies, often at personal cost and under the weight of stigma, which likely exacerbated health risks and social marginalization (13). The NSW policy reversal can be seen not only as a practical intervention but also as a symbolic correction, signaling that people who inject methadone are entitled to the same rights to health and safety as others.

Although people who inject methadone represent a small proportion of NSP clients, their inclusion in harm reduction represents an important step in making NSP services equitable. Harm reduction interventions need to extend to all groups, regardless of size, particularly to those facing structural barriers and stigma. In other words, the right to health and safety should not be contingent on prevalence but on vulnerability and need (16, 23). By tailoring services to meet the needs of this population, KRC has addressed a longstanding gap in harm reduction provision.

### Limitations

This case study reports on service data at KRC and a small quality improvement survey. There are inherent limitations in interpreting routinely collected data. Our approach of triangulating different data sources across KRCs NSP database, order records, and a brief client survey, aimed to mitigate these limitations.

During routine data collection, missing values may occur (for example, 5.2% of dispensing occasions were to people whose last drug used was not known). This may bias our findings (e.g., some individuals in this group may have used methadone as their last drug). Similarly, ‘butterfly packs’ may have been obtained by people who injected a different drug last time. Dispensing occasions of ‘butterfly packs’ were not captured in this study because the NSP reporting system did not have a functionality to record this new equipment. In the NSP database, 9,634 combined needle and syringe packs were distributed to people who reported methadone as their last drug injected. This represents approximately two-thirds of the ‘butterfly’ equipment ordered for distribution, suggesting that there may have been up to 30% more visits related to methadone injecting than what was captured by the NSP database.

Regarding the survey presented in this study, the sample size was small. This, potentially, limits the significance of findings – the association between increased attendance and using butterflies every time people inject or very often, may have been only marginally significant due to the low sample size. Indeed, had there been more participants in the survey, the study would have more statistical power to assess associations between the self-observed improvements in safety and health and increased program attendance.

NSP staff attempted to distribute the survey to all clients known to obtain ‘butterfly’ equipment from KRC. As such, the sample size of eighteen appears proportionate to the 125 dispensing occasions where the last drug injected was methadone (this would yield about seven attendances per participants over the 24 months post-implementation). Participant characteristics were not collected in the survey but may correspond to those recorded in the NSP database for occasions of service to people who last used methadone.

## Conclusions

Equipment for intravenous administration of methadone can be safely and efficiently distributed by NSP programs, to decrease health risks associated with injecting methadone and improve engagement of people who inject methadone with support services. The reintroduction of winged infusion sets and larger-volume syringes into harm reduction programs demonstrates how policies and service adaptations can promote greater inclusivity, ensuring that people who inject methadone are equitably supported.

## Data Availability

The data analyzed in this study is subject to the following licenses/restrictions: Restrictions from the Governance of South Eastern Local Health District. Requests to access these datasets should be directed to vendula.belackova@health.nsw.gov.au.
